# Acupuncture for ocular myasthenia gravis: A protocol for systematic review and meta-analysis

**DOI:** 10.1097/MD.0000000000019901

**Published:** 2020-04-24

**Authors:** Di Zhang, Song Jin, Leixiao Zhang, Lin Chen, Fanrong Liang

**Affiliations:** a Hospital of Chengdu University of Traditional Chinese Medicine; b Hospital of Chengdu University of Traditional Chinese Medicine; c Chengdu University of Traditional Chinese Medicine; d Chengdu Shuangliu Hospital of Traditional Chinese Medicine; e The 3rd Teaching Hospital, Chengdu University of Traditional Chinese Medicine, Chengdu, Sichuan, China.

**Keywords:** Ach, acupuncture, ocular myasthenia gravis, randomized controlled trial

## Abstract

**Background:**

The aim of this systematic review with meta-analysis is to determine the efficacy and security of acupuncture in treatment of ocular myasthenia gravis and find out whether or not the quick short-term efficacy of acupuncture exists.

**Methods:**

The following electronic databases will be searched by 2 independent reviewers: PubMed, Cochrane Library, EMBASE, Springer, China National Knowledge Infrastructure, Wanfang, and Chinese Biomedical Literature Database. All randomized controlled trials on acupuncture for ocular myasthenia gravis published in electronic databases from inception to March 1, 2020, with language restricted in Chinese and English will be included in the study.

Methodologic quality is assessed by 2 blinded reviewers independently screen and score the articles using the PEDro scale and the Cochrane Collaboration risk of bias tool. A meta-analysis was performed when there is sufficient clinical homogeneity in at least 2 studies. The Grading of Recommendations Assessment, Development and Evaluation approach is used to rate the body of evidence in each meta-analysis. When the quantitive evaluation is not available, a qualitative description of the results of single study is provided.

**Results:**

An evidence of variety of acupuncture treatment methods for treating ocular myasthenia gravis will be illustrated using subjective reports and objective measures of performance. The primary outcomes consisted of effective rate, MGFA PIS, QMG, and MG-composite. Secondary outcomes involve clinical absolute and relative score, titers of AchR antibodies, and the side effects. The treatment frequency and courses will be measured.

**Conclusion:**

This protocol will present the evidence of whether acupuncture is an effective and safe intervention for ocular myasthenia gravis.

**Trial registration number:**

CRD42019141325

## 1 Introduction

Acquired myasthenia gravis (MG), which is characterized by a typical pattern of fatigable muscle weakness, is the most common primary disorder of neuromuscular transmission and results from the binding of autoantibodies to components of the neuromuscular junction, most commonly the acetylcholine receptor (AChR). The incidence ranges from 0.3 to 2.8 per 100,000 and it is estimated to affect more than 700,000 people worldwide.^[1,2]^ According to an international consensus guidance for the management of MG published on Neurology in 2016, definitions of MG were developed into 5 classes, such as Remission, Ocular MG, Impending Myasthenic Crisis, Manifest Myasthenic Crisis, and Refractory MG. By this clinical classification, any kind of ocular muscle weakness of eye closure without abnormal strength of all other facial, bulbar, and limb muscles were defined as ocular MG. kinds of treatment approaches were conducted all over the world, since the varieties of MG, kinds of treatment approaches were conducted all over the world: symptomatic and immunosuppressive (IS) treatments, intravenous immunoglobulin (IVIg) and plasma exchange (PLEX), management of impending and manifest myasthenic crisis, thymectomy, juvenile MG, MG associated with antibodies to muscle specific tyrosine kinase (MuSK-MG), and MG in pregnancy. However, because of various adverse effects such as nausea, vomiting, diarrhea, and other gastrointestinal symptoms for symptomatic treatment, or hypertension, hyperglycaemia, overweight, and other endocrine system diseases for IS treatment, or myelosuppression, liver, renal toxicity for IVIg, and bleeding, secondary infection for PLEX, and so on, along with applicable limitation such as high cost, hospital scale and level, restrict applicable crowd, and so forth, the extensive promotion and application of above treatments seems to be difficult, especially for MG patients in remote regions and in poverty.^[3]^

Acupuncture as a non-pharmacological, convenient, and low-cost therapy has been attracting increasing attention from medicine circles in the world. Nowadays, acupuncture is often used for ocular MG patients in eastern countries.^[4–37]^ Several researches have confirmed that acupuncture could improve ocular MG symptoms by increasing acetylcholine potentials (AchP) and small endplate potential (MEPP) amplitude to a certain extent,^[38]^ or achieve therapeutic effects by changing the affinity between acetylcholine receptor (AchR) and acetylcholine (Ach) or acetylcholine receptor antibody (AchRab) on the postsynaptic membrane of a neuron-muscle junction.^[39]^ However, most of these researches are small samples of randomized clinical trials or even non-RCTs; the efficiency of acupuncture for ocular MG is still open to question.

As far as we know, there are a protocol and a SR of acupuncture for MG published in order in 2019 with a retrieval deadline before July 31, 2018, and September, 2019.^[40,41]^ However, several more relative RCTs have been published just later,^[42–46]^ and there is no analysis result of MG types in both reviews despite of the known facts that acupuncture is more used in treatment of ocular MG and the treatment courses could be short and quick. Thus, the conclusion of “acupuncture could be beneficial for MG patients only with long-term therapy” of the review seems to be controversial. Therefore, we feel it necessary to evaluate the acupuncture efficacy only for ocular MG, which is the mildest and most common type of MG in the world, and we are actually looking forward to a new different conclusion which could provide a valid evidence to the short-term efficacy of acupuncture for ocular MG compared to the published review. Thus, in this paper, we will systematically review and critically evaluate the published RCTs by comparing acupuncture with various types of control interventions to determine the effectiveness and security for specific ocular MG. Different conclusions can be drawn, and the meta-analysis of RCTs will provide sufficient data.

## 2 Methods

**Figure 1:**
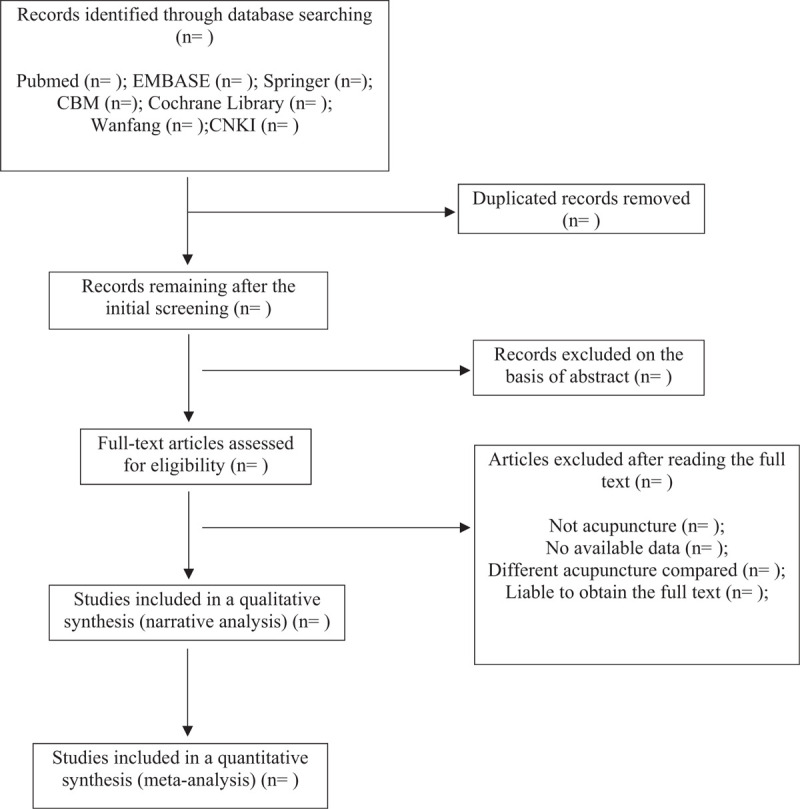
Flow diagram of studies identified.

The protocol of this systematic review has been registered on PROSPERO with a registration number of CRD42019141325. We will develop the protocol strictly according to the guidelines of Preferred Reporting Items for Systematic Reviews and Meta-Analyses protocols (PRISMA-P)^[47]^ (Fig. 1).

### 2.1 Search strategy

Relevant databases including PubMed, Cochrane Library, EMBASE, Springer, China National Knowledge Infrastructure, Wanfang, and Chinese Biomedical Literature Database will be searched. All RCTs published in electronic databases from inception to March 1, 2020, with language restricted in Chinese and English will be included in this review study. The Medical Subject Headings (MeSH), text words, and word variants for “ocular myasthenia gravis” and “acupuncture” or “acupoint” or “meridian” or “electroacupuncture” or “transcutaneous electrical nerve stimulation” or “acupoint catgut embedding” or “auriculotherapy” or “acupoint injection” or “fire needle” or “needle knife” or “superficial needling” or “acupressure” or “cupping jar” or “moxibustion” or “acupoint application” or “abdominal acupuncture” or “bleeding” are used and combined in the searches. The primary selection process is shown in PubMed search strategy (Table 1).

**Table 1:**
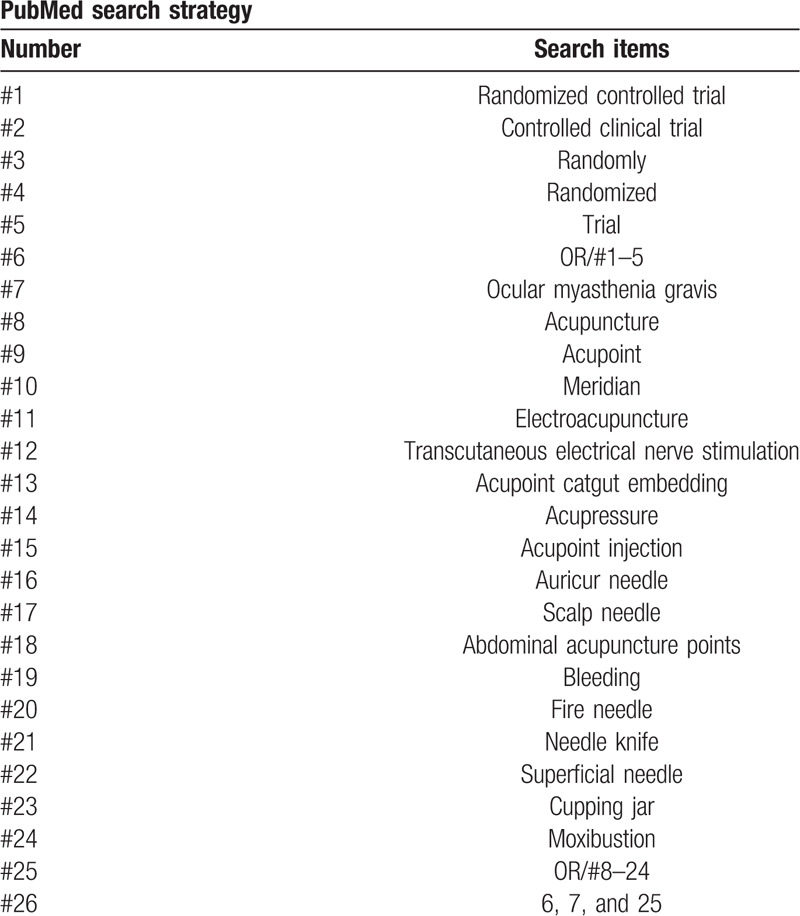
Search strategies applied to this review.

### 2.2 Inclusion and exclusion criteria

#### 2.2.1 Types of study

To evaluate the curative effects of acupuncture on ocular MG, this review is confined to RCTs comparing any form of acupuncture with a control group, which contained drug, no treatment, placebo, diet, and exercise therapy. It is deemed a randomized study if the trial stated the “randomization” phrase, and the blinding is not restricted. The language will be limited to Chinese and English. The animal mechanism studies, case reports, self-pre- and post-control, or non-RCTs are excluded.

#### 2.2.2 Types of participants

It included the participants with no limitation of age. The MG selected is only type I with the lesion confined to the extraocular muscle, and no more muscle groups were affected within 2 years according to the ICD-11 MMS (2018 version) and the Chinese Guidelines for Diagnosis and Treatment of Myasthenia Gravis (2015).^[48,3]^ The definitions of ocular MG are included. Patients with acute medical conditions or pregnancy are excluded.

#### 2.2.3 Types of intervention

The review comprises clinical trials with the treatment of acupuncture. We will study the types of acupuncture including acupuncture, electroacupuncture, transcutaneous electrical nerve stimulation, acupoint catgut embedding, acupressure, cupping jar, moxibustion, auricular acupuncture, scalp needle, abdominal acupuncture, bleeding, acupoint injection, fire needle, needle knife, and superficial acupuncture. Studies to compare the effect of different acupuncture therapies will be excluded.

#### 2.2.4 Types of outcome measures

The primary outcomes consisted of effective rate, Myasthenia Gravis Foundation of American Post Intervention Status (MGFA PIS), Quantitative MG Scale (QMG), or MG-Composite. Secondary outcomes include clinical absolute and relative score, titers of AchR antibodies. SF-36 or MG-QOL, treatment frequency and courses, as well as side effects, such as pneumothorax, bleeding, serious discomfort, subcutaneous nodules, and infection, will also be recorded.

### 2.3 Data collection and analysis

Two authors independently scanned the titles and abstracts. The studies that satisfied the inclusion and exclusion criteria are retrieved for full-text assessment. The extracted data included the first author, publication year, RCT design, country, sample size, number of males and females, mean age of the population, blind method, randomization, interventions (including the frequency and duration of interventions), primary outcome measures, follow-up time, results, and conclusions. The results regarding the outcome measures are extracted in the form of mean and standard deviation data. For cross-over trials, the summary data are used as if they had been derived from parallel trials. For trials with more than 2 intervention groups, the experimental group was compared with the control group by combining the data of all relevant control groups. The remaining discrepancies in data extraction are resolved after the discussion between the 2 reviewers. A third reviewer adjudicated when necessary.

#### 2.3.1 Data extraction and management

The extracted information includes descriptions of studies, characteristics of participants, interventions of both observation group and control group, quality, randomization, allocation concealment and blinding methods, outcome measures, main outcomes, adverse effects, duration of follow-up, type and source of financial support, and the Standards for Reporting Interventions in Controlled Trials of Acupuncture (STRICTA) checklist.

#### 2.3.2 Assessment of risk of bias and reporting of study quality

Risk of bias is used to evaluate the quality of study with the Cochrane Collaboration's risk-of-bias assessment method and complete the STRICTA checklist for the included studies. The decision of risk is made by 2 reviewers (ZD and ZLX). If inconsistent results appear, the final decisions will be made by the third author (JS). For missing or ambiguous data, we will try to contact the author as possible, and for duplicate publication we only select the original.

#### 2.3.3 Measures of treatment effect

Mean differences (MDs) with 95% confidence intervals (95% CIs) will be used to analyze continuous data. Other forms of data will be changed into MD values. Risk ratio with 95% CIs will be used to analyze dichotomous data. If significant heterogeneity is detected, a random-effects model will be used.

#### 2.3.4 Unit of analysis issues

The analysis will focus on patients in randomized studies. If more than 1 objective is used, we will conduct separate multiple meta-analyses for each treatment objective. If multiple acupuncture control groups are included, pooled analyses of the control groups against the intervention group will be used.

#### 2.3.5 Management of missing data

There are missing or incomplete data for the primary results; we will contact the corresponding authors for the missing data. If the missing data cannot be obtained, it will be excluded from analysis.

#### 2.3.6 Assessment of heterogeneity

Review Manager (version 5.3, the Nordic Cochrane Centre, Copenhagen, Denmark) is applied to assess curative effect and publication bias. Forest plot is used to illustrate the relative strength of curative effect. Meanwhile, the funnel plot will picture the publication bias visually as the number of trials is more than 10. If significant heterogeneity is detected, a random-effects model will be used.

#### 2.3.7 Assessment of reporting biases

Funnel plots is used to assess reporting biases. If funnel plot asymmetry is detected, the reasons will be analyzed.

### 2.4 Data synthesis

#### 2.4.1 Narrative analysis

We may conduct narrative synthesis if meta-analysis is not appropriate (e.g., incidence of adverse events of acupuncture).

#### 2.4.2 Meta-analysis

RevMan V.5.3 will be employed for data analysis when meta-analysis is possible. The MD with 95% CIs will be used to assess continuous outcomes, while the RR with 95% CIs will be used for dichotomous data. If I^2^ < 50%, the RR and MD will be calculated by a fixed-effects model. If I^2^≥50%, a random-effects model, will be used to synthesis the data and subgroup analysis or sensitivity analysis will be conducted to explore the causes of heterogeneity including clinical or methodological reasons.

#### 2.4.3 Subgroup analysis

A subgroup analysis will be performed according to control intervention and different outcomes.

#### 2.4.4 Sensitivity analysis

A sensitivity analysis will be performed according to the following criteria: sample size, heterogeneity qualities, and statistical model (random-effects or fixed-effects model).

### 2.5 Grading the quality of evidence

The Grading of Recommendations Assessment, Development and Evaluation (GRADE) guidelines will be used to assess the quality of evidence for outcomes. The strength of the body of evidence will be graded into 4 levels including very low, low, moderate, or high.

### 2.6 Ethical approval and dissemination

There is no requirement of ethical approval in this protocol for no patients being involved, so that no individual privacy will be under concerns. The results of this review will be in print or disseminated by electronic copies.

## 3 Discussion

Acupuncture plays a role of treatment by a very thin needle with broader point that the therapist pushes through the skin to stimulate the trigger points. The resulting larger sample size and consistent presentation of data will allow additional analyses to explore patient-level heterogeneity in treatment outcomes and prognosis of MG. The results of this review indicate that multiple protocol-related variables may influence outcomes.

A wide variety of acupuncture protocols are used in the RCT's reviewed, which complicates comparison of study outcomes and possibly jeopardizes the external validity of this review. Therefore, multiple outcome measures should be explored per study, with measures over time, in order to better understand the potential benefits of acupuncture and guide clinical decision-making.

Results are limited by the quality and sample size of English/Chinese articles. Access to new, unpublished data may not be available by the time this review is submitted; therefore, the results presented here may be influenced by publication bias.

## Author contributions

ZD conceived this study and developed the first frame of this manuscript. ZD and ZLX drafted the manuscript. ZD, CL, and ZLX performed data collection, JS revised the manuscript, and LFR provided funding support. All authors were involved in the design, gathering of information, data analysis, write up, and final edits.

## Author contributions

**Conceptualization:** D. Zhang.

**Methodology:** L.X. Zhang, D. Zhang.

**Software:** L. Chen.

**Supervision:** S. Jin.

**Validation:** F.R. Liang.

**Writing:** D. Zhang, S. Jin.
